# Asymptomatic ALCAPA with Preserved Myocardial Function in a 3-Year-Old Child

**DOI:** 10.1007/s00246-025-04012-2

**Published:** 2025-09-08

**Authors:** C. Leclercq, F. Kaladji, J. P. Vallée, T. Nalecz, T. Sologashvili, M. Beghetti, J. Wacker

**Affiliations:** 1 Pediatric Cardiology Unit, University Hospital of Geneva, Geneva, Switzerland; 2 Radiology Department, University Hospital of Geneva, Geneva, Switzerland; 3 Pediatric Cardiac Surgery Unit, University Hospital of Geneva, Geneva, Switzerland

**Keywords:** ALCAPA, Late presentation, Myocardial injury

## Abstract

Anomalous origin of the left coronary artery from the pulmonary artery (ALCAPA) is a rare congenital anomaly. Its clinical course is typically severe in infancy, leading to left ventricular ischemia, cardiogenic shock, and high mortality without surgical intervention.

We describe a rare case of a 3-year-old girl diagnosed with ALCAPA, showing extensive right-to-left collaterals, preserved left ventricular function, and minimal myocardial injury.

## Case Report

A 3-year-old girl from Mali was referred by an NGO for surgical management of ALCAPA. The diagnosis was initially made at one year of age during the evaluation for recurrent respiratory infections, poor weight gain, and a cardiac murmur.

Upon presentation to our institution, she appeared in good general condition, without physical limitations or chest pain. Physical examination revealed a 2/6 systolic murmur along the left sternal border. ECG was nonspecific, showing no Q waves or ST-segment changes.

**Fig. 1:**
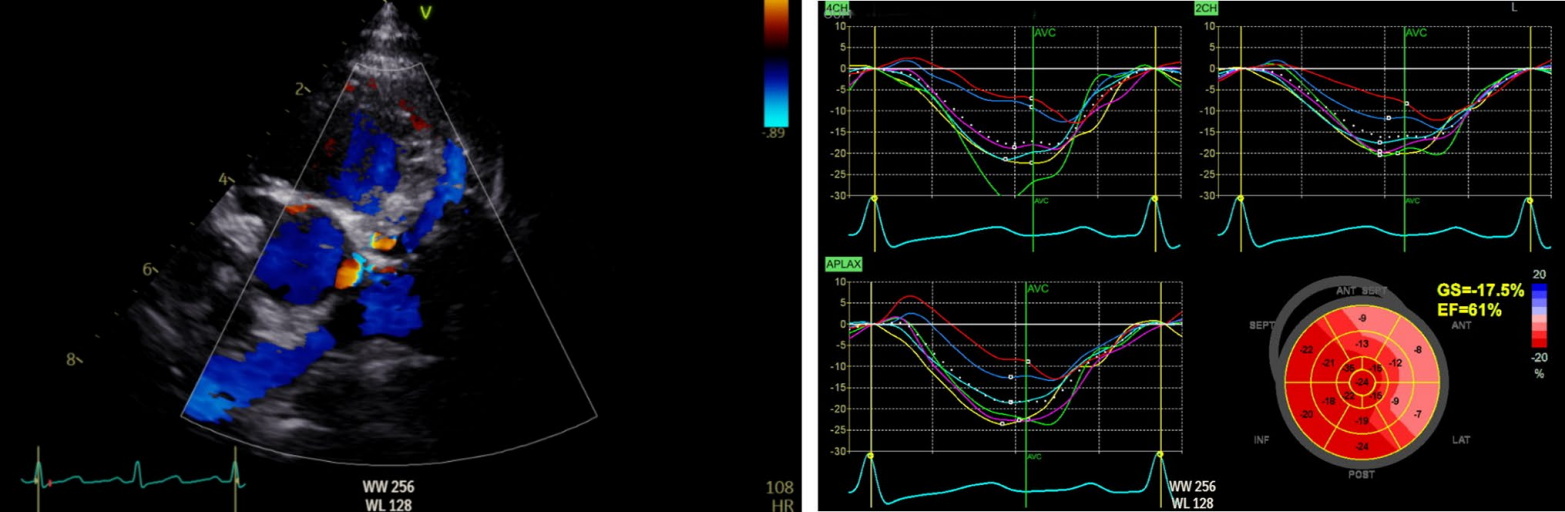
On the left panel, parasternal short axis with color doppler demonstrating a dilated right coronary artery (RCA) and the left coronary artery arising from the posterior side of the pulmonary trunk with retrograde flow. On the right panel, Global Longitudinal Strain subtle regional abnormalities in the anterolateral territories

Transthoracic echocardiography confirmed the diagnosis of ALCAPA, showing retrograde flow from the left coronary artery into the pulmonary trunk, originating from its posterior side. No proximal stenosis was present. The right coronary artery was severely dilated (Z-score + 6.8), and numerous tortuous vessels were visualized across the myocardial surface, suggestive of extensive collateral formation. The left ventricle was normal in size with preserved systolic function (LVEF 61%, Simpson biplane), no segmental wall motion abnormalities, and a competent mitral valve. Global longitudinal strain (GLS) was borderline at −17.5%, with subtle regional abnormalities in the anterolateral territories (Fig. 1).

**Fig. 2:**
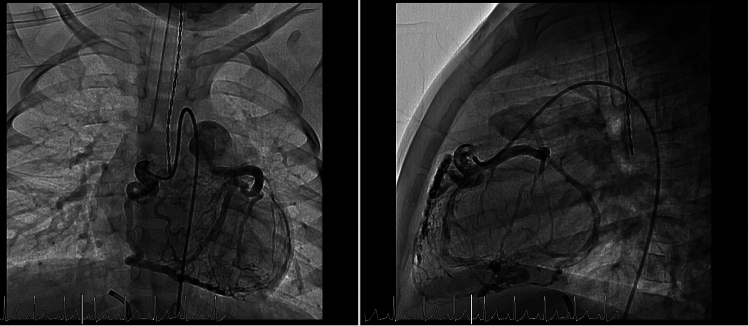
Angiography from the right coronary artery demonstrating tortuous dilated vessels connecting to the left artery to the pulmonary trunk

Cardiac catheterization was performed to further assess coronary anatomy and collateral circulation. Angiography of the right coronary artery demonstrated opacification of the left coronary system via extensive collaterals (Fig. 2). Left ventricular end-diastolic pressure was elevated at 17 mmHg, while pulmonary artery pressures were normal.

Cardiac MRI showed no delayed contrast enhancement and no regional wall motion abnormalities. The Qp/Qs ratio was estimated at 1.1–1.2.

**Fig. 3:**
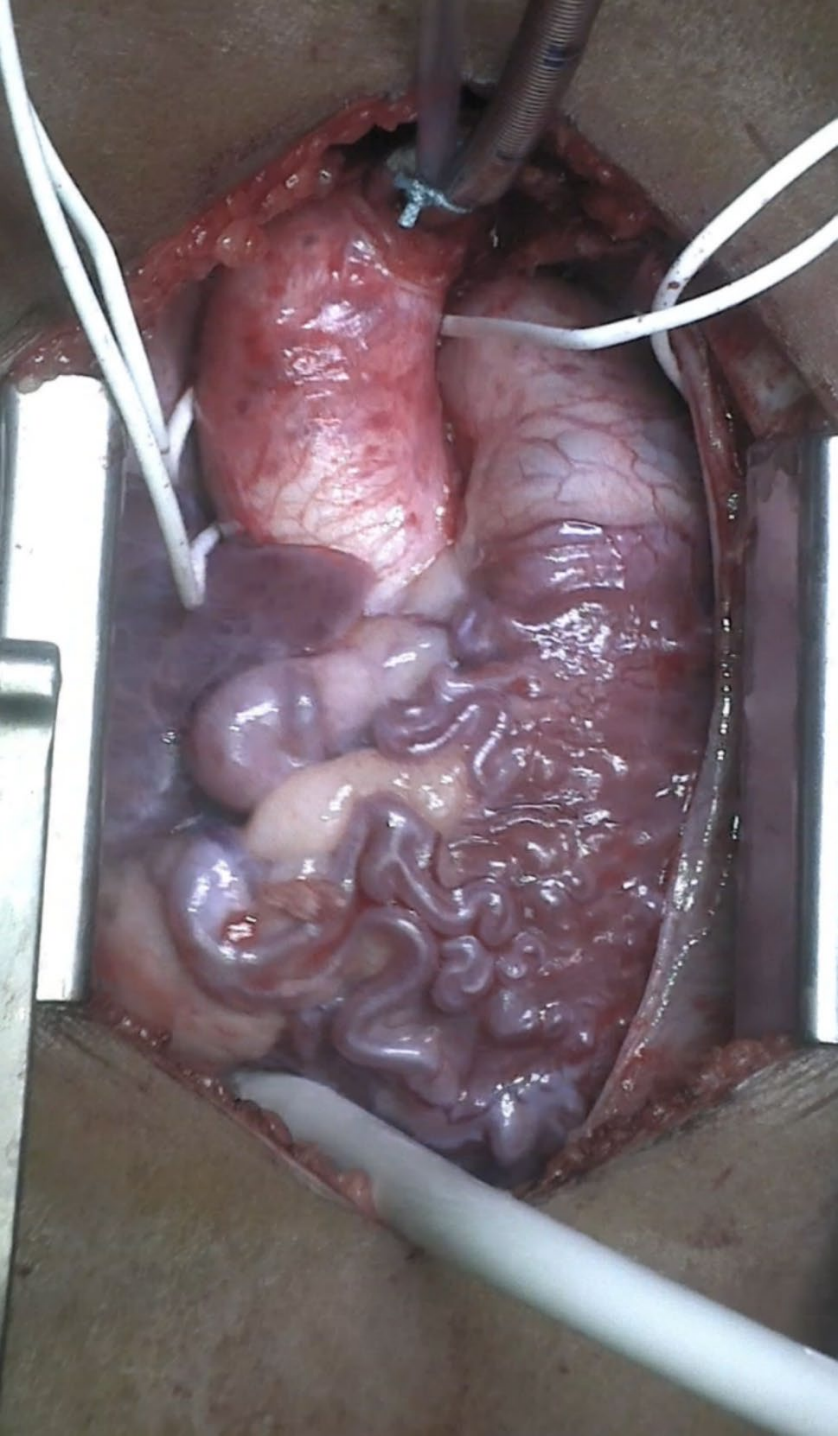
Peroperative view of the extensive right to left coronary collaterals

The patient underwent complete surgical repair via coronary translocation to the ascending aorta (Fig. 3). The pulmonary artery sinus defect was reconstructed using an autologous pericardial patch. Intraoperative echocardiography showed restoration of antegrade flow in the left coronary artery and normal LV function. The postoperative course was uneventful, and she was discharged on postoperative day 5.

## Discussion

This case represents the rare subset of approximately 10% of ALCAPA patients who survive beyond infancy without early surgical correction [1]. Remarkably, our patient presented with minimal myocardial injury despite ongoing coronary steal and a significant left-to-right shunt.

Myocardial perfusion in this patient was likely maintained through the development of extensive right-to-left coronary collaterals, which effectively compensated for the abnormal coronary anatomy. Nevertheless, the mildly reduced global longitudinal strain and elevated left ventricular end-diastolic pressure suggest prior episodes of subendocardial ischemia, likely occurring before the collateral network had fully matured.

Interestingly, late-presenting ALCAPA cases with preserved myocardial function often show protective features such as proximal LCA stenosis, limiting coronary runoff. In this case, no such protective stenosis was present, making the extent of myocardial preservation particularly striking. Female preponderance of late presentation has been found suggesting gender differences in compensatory changes (2).

Without surgical intervention, the natural progression in this patient would likely have been toward chronic subendocardial ischemia and gradual deterioration of left ventricular function. Even in cases with minimal symptoms and preserved function, surgical correction of late-presenting ALCAPA is indicated to prevent long-term complications such as heart failure, malignant arrhythmias, and sudden cardiac death (3).

This case highlights the remarkable adaptive capacity of the pediatric myocardium in the setting of ALCAPA, as demonstrated in this asymptomatic 3-year-old child.

## Data Availability

No datasets were generated or analysed during the current study.
