# Correction to: Asymptomatic ALCAPA with Preserved Myocardial Functionin a 3‑Year‑Old Child

**DOI:** 10.1007/s00246-025-04109-8

**Published:** 2025-11-17

**Authors:** C. Leclercq, F. Kaladji, J. P. Vallée, T. Nalecz, T. Sologashvili, M. Beghetti, J. Wacker

**Affiliations:** 1 Pediatric Cardiology Unit, University Hospital of Geneva, Geneva, Switzerland; 2 Radiology Department, University Hospital of Geneva, Geneva, Switzerland; 3 Pediatric Cardiac Surgery Unit, University Hospital of Geneva, Geneva, Switzerland

**Correction to: Pediatric Cardiology**

10.1007/s00246-025-04012-2

In this article, Figure 1 appeared incorrectly and have now been corrected in the original publication. For completeness and transparency, the old incorrect version and corrected version are displayed below:

**Incorrect Figure 1**


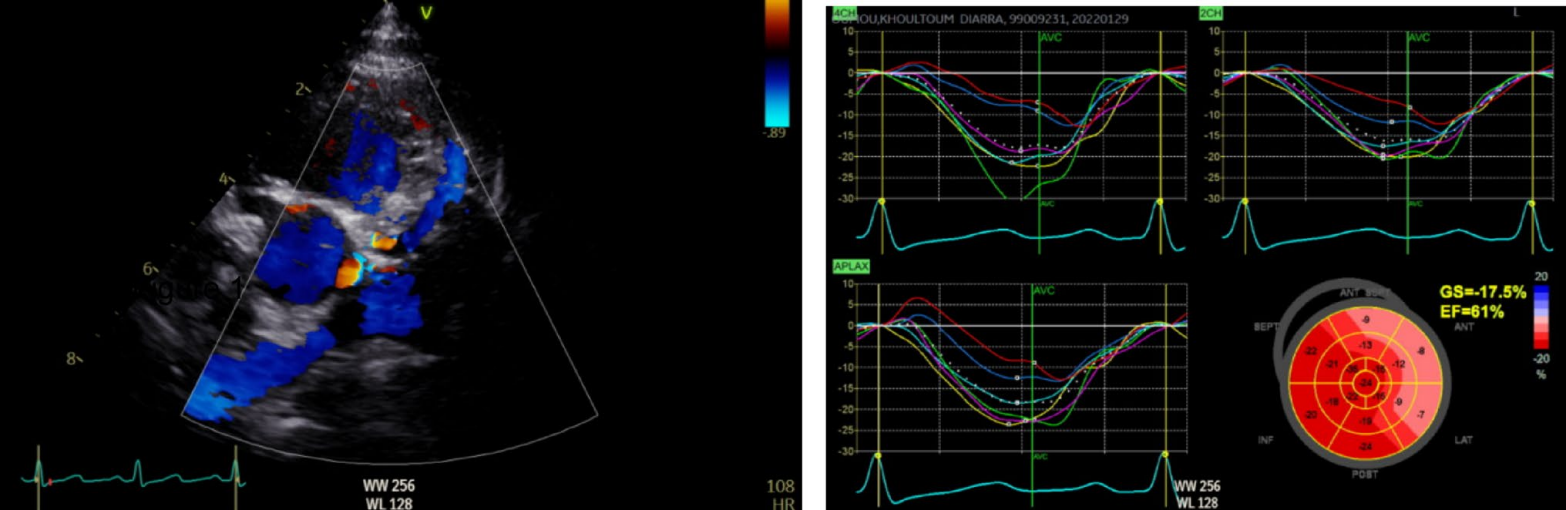


**Correct Figure 1**


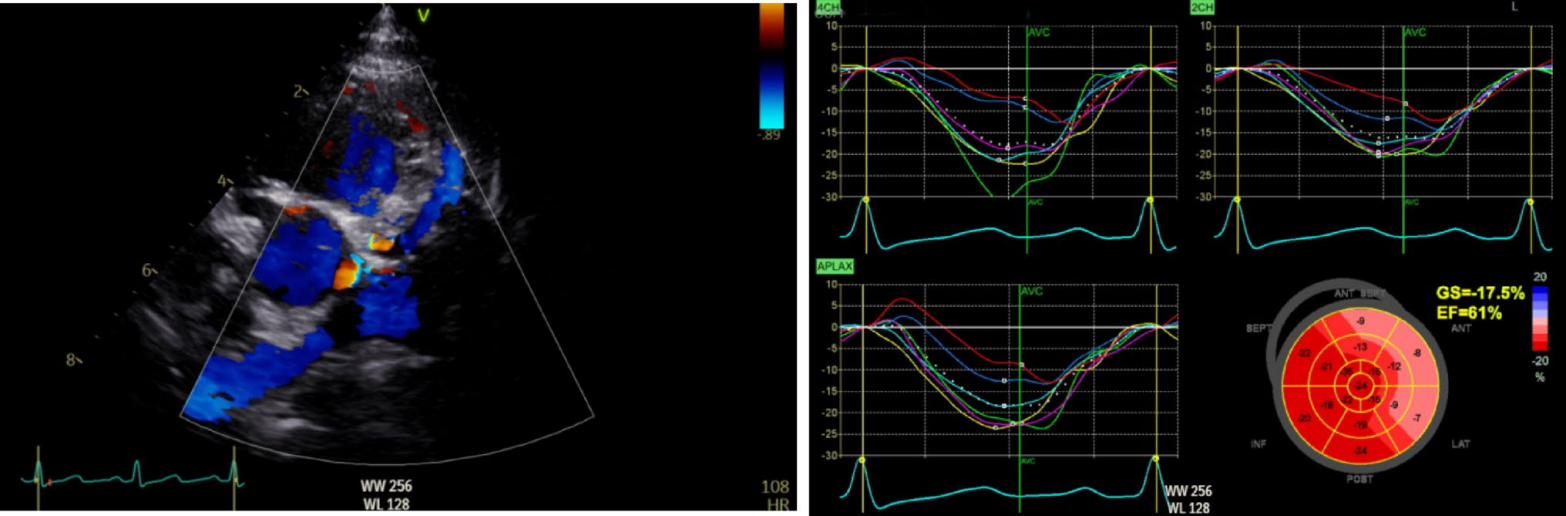


The original article has been corrected.

